# Characteristics of precipitation changes during tropical cyclone processes in China from 1980 to 2019

**DOI:** 10.1038/s41598-024-64252-9

**Published:** 2024-06-13

**Authors:** Guangran Zhai, Wei Xu, Peng Su, Lianjie Qin, Xinli Liao

**Affiliations:** 1 Key Laboratory of Environmental Change and Natural Disaster of Ministry of Education, Beijing Normal University, Beijing, 100875 China; 2 State Key Laboratory of Earth Surface Processes and Resource Ecology, Beijing Normal University, Beijing, 100875 China; 3 Academy of Disaster Reduction and Emergency Management, Ministry of Emergency Management and Ministry of Education, Beijing Normal University, Beijing, 100875 China; 4 Faculty of Geographical Science, Beijing Normal University, Beijing, 100875 China; 5 Institute for Public Safety Research, Department of Engineering Physics, Tsinghua University, Beijing, 100084 China; 6 School of Safety Science, Tsinghua University, Beijing, 100084 China

**Keywords:** Tropical cyclone precipitation, China, Regional analysis, Spatiotemporal distribution, Process precipitation, Natural hazards, Atmospheric science

## Abstract

Tropical cyclones (TCs) and their associated intense rainfall are among the most significant natural disasters. Exploring the characteristics of tropical cyclone precipitation (TCP) has always been a challenging issue in TC research. This study utilized the TC track data from the International Best Track Archive for Climate Stewardship and precipitation data from the multi-source weighted-ensemble precipitation covering the years 1980–2019, to examine shifts in precipitation rates and peak precipitation levels before and after TC landfall. The results highlight several key findings: (1) Precipitation during the TC landfall process is relatively stable beforehand but tends to decrease slightly after landfall. Generally, the maximum precipitation occurs during the landfall. (2) From 1980 to 2019, the rate of precipitation changes before landfall has significantly increased. Conversely, after the year 2000, the rate of precipitation changes after landfall has significantly decreased. (3) Over the past 40 years, while peak precipitation levels of landfalling TCs have remained relatively constant, the total precipitation has shown an increasing trend, particularly in regions like the main island of Hainan, southern Zhejiang, and Shanghai, which are characterized by high peak precipitation. The results help clarify the TC processes and provide reference points for parameter selection in regional TCP modeling.

## Introduction

Approximately one-third of global tropical cyclones (TCs) are generated annually in the Northwest Pacific, and the southeastern coastal areas of China are among the regions where are most severely impacted by TCs. In addition to strong winds, heavy rainfall is also a significant factor contributing to the destructive nature of TCs^1–6^. Tropical cyclone precipitation (TCP) often leads to secondary hazards such as urban waterlogging, flash floods, landslides, and mudslides, which result in significant economic losses and casualties^4,7^. For example, in 2006, the Severe Tropical Storm Bilis, which made landfall in Fujian, China, resulted in massive losses due to flash floods and mudslides triggered by heavy rainfall^8^. Hurricane Harvey, which made landfall in Houston, Texas, in 2017, brought over 1 m of precipitation and caused severe flooding^4^. In recent years, the number of TCs making landfall in China has decreased^9,10^, but the TC intensity has increased significantly^11^. Due to global warming, it is expected that future TC intensity will continue to increase^12–17^, and the risk of TCP might also increase. Exploring the characteristics of TCP can aid in understanding the process of TC disasters and provide a scientific basis for assessing TC loss.

Currently, many researches on TCP focused on analyzing the characteristics of TCP changes and simulating TCP. Among the existing studies on the characteristics of TCP changes, research has mainly focused on spatial distribution changes in TCP, as well as temporal changes in the number or intensity of TCP^18–23^. And in temporal change analysis, studies usually focus on monthly and annual TCP scale changes. For example, Tan et al.^24^ have used daily resolution precipitation data to analyze the annual TCP variation trends in four basins from 1983 to 2019. Su et al.^25^ have analyzed the annual variation of extreme TCP in southeastern China using daily resolution station precipitation data. Even though some studies use hourly resolution precipitation data, the conclusions mostly focus on the analysis of large-scale changes in TCP^26–30^. For example, Guzman et al.^19^ and Tu et al.^31^ used higher resolution TRMM precipitation data (with a time resolution of 3 h) to analyze changes in global TCP rates, but also focused on annual trends. Current researches have paid less attention to TCP changes during TC landfall process. Some studies also focus on the hourly TCP changes. Zhang and Chen^32^ have analyzed the TCP characteristics of Typhoon Nida in Guangzhou in 2016 utilizing 1-h resolution precipitation data estimated by Doppler radar. Gao et al.^8^ have used hourly precipitation observation data to analyze the characteristics of three precipitation stages during the severe tropical storm Bilis in 2006. However, this type of research focused on analyzing the TCP changes during a single TC event. And insufficient analysis of the general patterns of hourly TCP changes.

The main factors commonly used in TCP simulations include the TC track, storm size, humidity, and vertical wind shear at a certain moment^33^. The most basic issue is to find the quantitative relationship between TCP and these parameters^34–36^. A correlation between the current TCP and the subsequent TCP patterns is also found^37^. However, most TCP simulations do not consider this correlation, mainly due to the difficulty in finding parameters that are closely related to precipitation changes during the TC process.

The precipitation of a TC varies throughout its development. In the sea, the intensity of TCP is positively correlated with the intensity of TC^37–40^. The intensity of a TC decreases due to terrain and other factors after it makes landfall^41,42^, and the TCP also decreases. The impact of TCP on land is mainly concentrated within 24 h before landfall and 24 h after landfall^43^. However, there is a lack of detailed research on precipitation changes during the development and dissipation processes of TCs. Therefore, in response to the above two issues of insufficient attention to the TCP changes during TC processes and the lack of process related variables in TCP simulation, this study will quantify the changes in precipitation during the TC landfall process. In this study, TCP of each TC event in China from 1980 to 2019 were first extracted by using a fixed threshold method based on multi-source weighted-ensemble precipitation (MSWEP) data and TC track data. Then the precipitation within 24 h before landfall and 24 h after landfall of a TC was calculated and compared to analyze the changes in precipitation during the process of TC landfall. The result can help to understand TCP characteristics during the TC process, and can provide key parameters for TCP simulations and assessment.

## Data and methods

### Data

This study used two primary types of data: optimal track data for tropical cyclones and global precipitation data. The TC track data were sourced from the International Best Track Archive for Climate Stewardship (IBTrACS)^44^, which records tropical cyclone activity from the mid-nineteenth century to the present. IBTrACS updates its data every 3 h, comprehensively documenting key parameters such as the center location, maximum wind speed, and minimum pressure of each tropical cyclone from its formation to dissipation. To meet the specific needs of this research, data provided by the China Meteorological Administration (CMA) were selected, focusing particularly on the cyclone tracks that affected the southeastern coast of China from 1980 to 2019. A total of 327 tropical cyclone records were selected during this period, 83% of which reached tropical storm strength or higher. The wind field data is simulated using the IBTrACS dataset based on the Holland wind field model^45–47^. This model is an analytical model for the radial distribution of sea level pressure and wind in TCs, and has good performance in simulation results.

The precipitation data were derived from the multi-source weighted-ensemble precipitation (MSWEP) reanalysis dataset. This dataset is a high-resolution (0.1° grid and updated every 3 h) global precipitation product that covers precipitation events from 1979 to the present^48,49^. The dataset is a 3-h precipitation data records, the 0:00 precipitation represents the sum of precipitation from 0:00 to 3:00, and the 3:00 precipitation represents the sum of the precipitation from 3:00 to 6:00, and so on. Comparative studies of 22 gridded precipitation datasets globally have shown that MSWEP exhibits the best temporal correlation and overall performance in alignment with ground-based observations^50^, hence its selection as the precipitation data source for this study.

### TC landfall process

A TC generally begins to have an impact on land before its landfall. After landfall, the intensity of the TC weakens due to terrain obstruction, and its precipitation also gradually weakens and then disappears. The impact of TCP on land was mainly concentrated within 24 h before landfall and 24 h after landfall^43^. In this study, we define the 48–24 h before TC landfall and 24 h after TC landfall—as the TC landfall process. The characteristics of TC precipitation changes during this period is focused.

### TCP extraction

**Figure 1:**
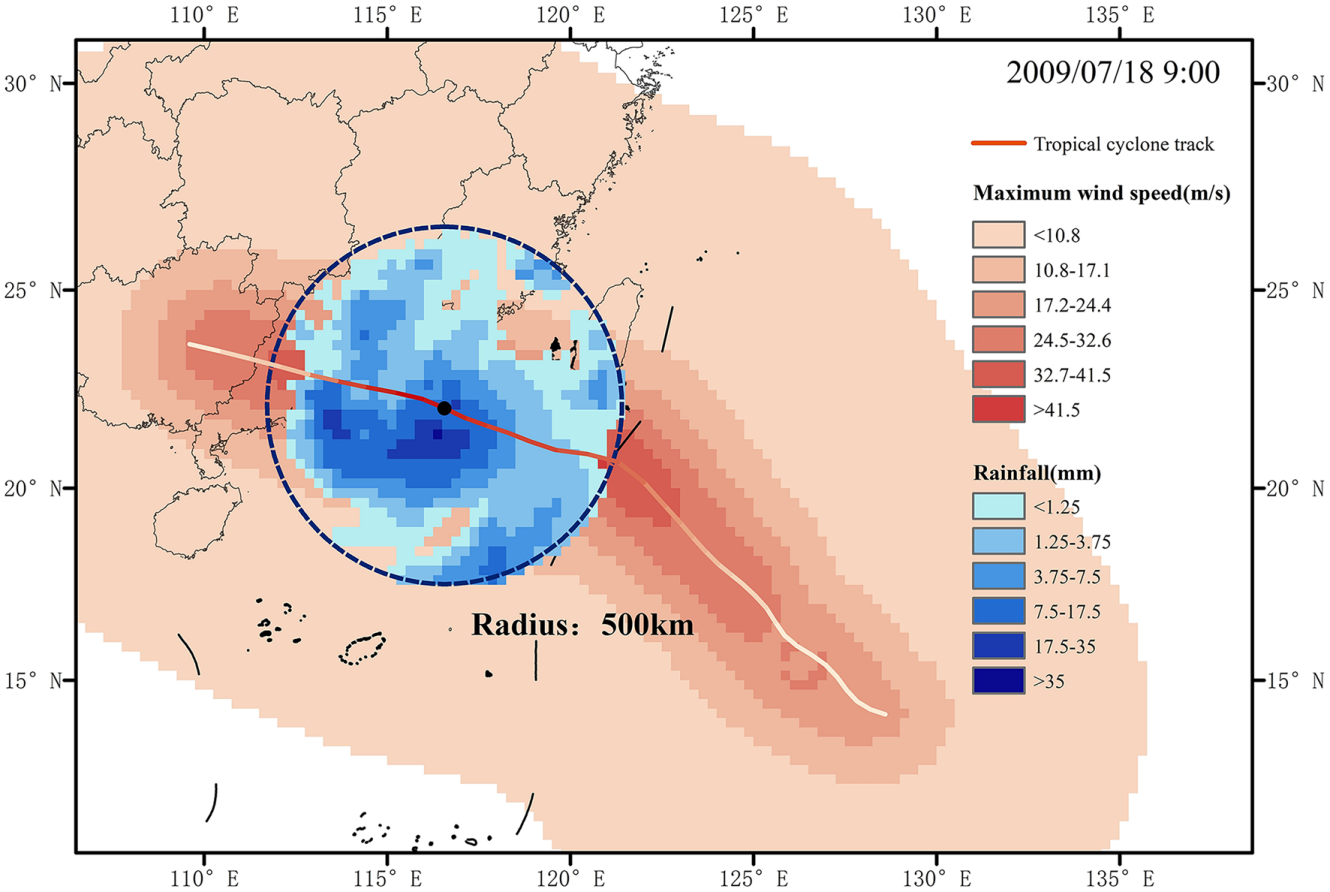
Example of Extracting TCP. The figure displays the maximum wind speed field (in red) of Typhoon Morakot in 2009, and the precipitation amount (in blue) at 9:00 on July 18, 2009 extracted using a fixed threshold (radius = 500 km) method. The wind field data is simulated using the Holland wind field model, while the precipitation data is obtained from the MSWEP dataset.

TCP generally refers to the rainfall observed within a specified radius from the center of a TC^19,30,51,52^. When the distance exceeds 450 km from the TC center, the average precipitation rate diminishes to less than 0.5 mm per hour^31^. Considering the impact of TCP outside 450 km, we expand the radius to 500 km for extracting TCP data. In addition, it is necessary to use a minimum precipitation intensity threshold to extract each continuous rain field. By referring previous studies^19^, we employed a precipitation threshold of 0.1 mm per hour. Figure 1 illustrates an example of TCP extracted from the MSWEP precipitation dataset.

**Figure 2:**
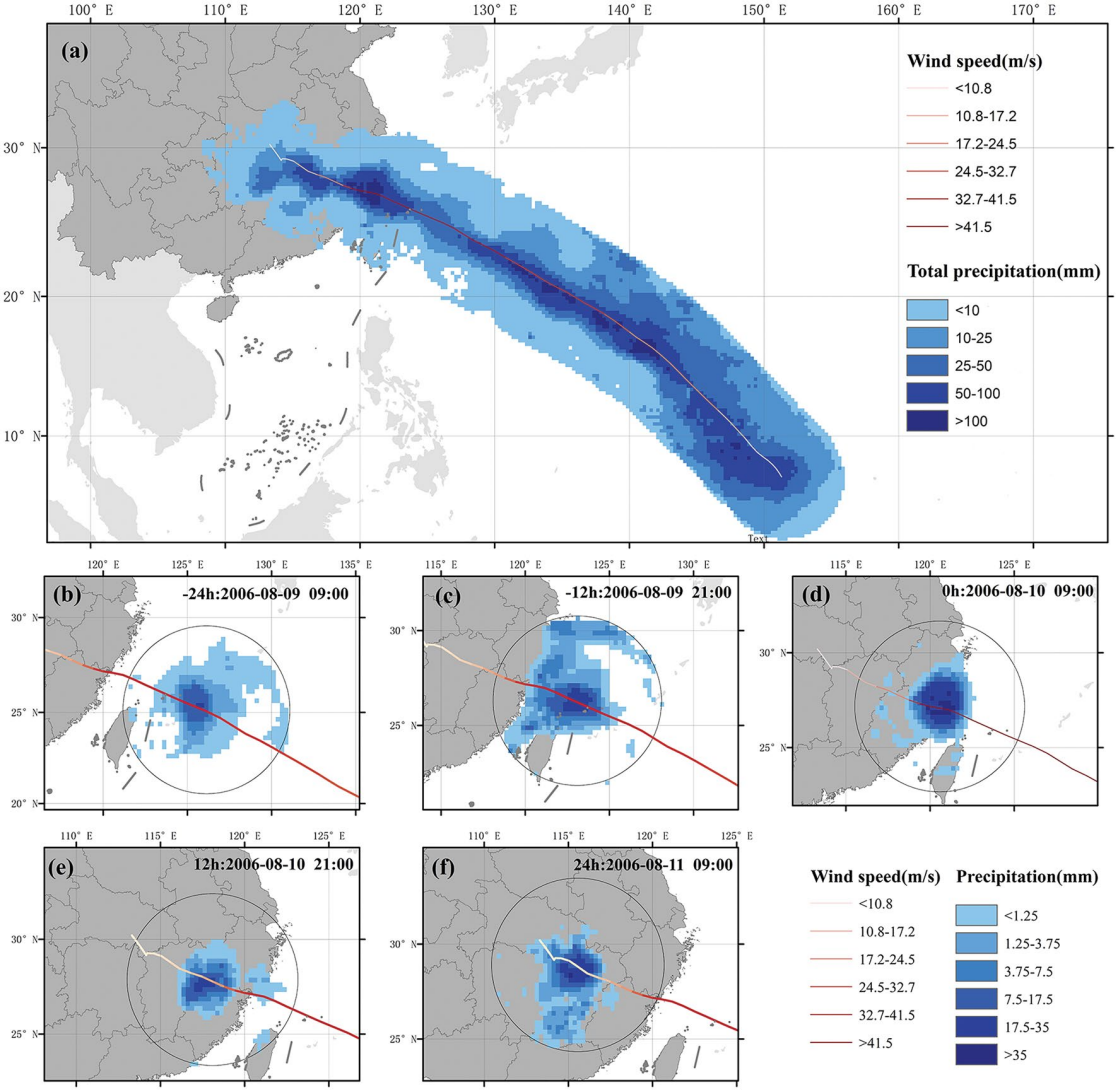
Characteristics of precipitation changes during Tropical Cyclone Saomai in 2006. (**a**) Cumulative precipitation during the TC process. (**b**) 24 h before landfall, (**c**) 12 h before landfall, (**d**) Moment of landfall, (**e**) 12 h after landfall and (**f**) 24 h after landfall.

Cumulative precipitation refers to the total amount of precipitation generated during the whole life of TC (namely from its formation to dissipation). Employing this method, the cumulative precipitation during the TC process can be extracted. Figure 2a shows the cumulative precipitation during the TC process of the 2006 tropical cyclone Saomai. The maximum cumulative precipitation produced during the development of Saomai reached 124.5 mm (Fig. 2a). Over the sea, TCs typically have higher precipitation intensities but have little impact on land (Fig. 2b,c). However, when a TC makes landfall, due to the uplift of the land, the TC containing water vapor will condense to form precipitation. As shown in Fig. 2, Typhoon Saomai had the largest amount of precipitation at the moment of it made landfall during its landfall process, with a maximum value of 43.8 mm (Fig. 2d). After landfall, the precipitation intensity of TCP exhibited a decreasing trend (Fig. 2e, f). Therefore, the maximum precipitation intensity after landfall usually occurs around the moment of landfall. In the following, maximum precipitation refers to the maximum precipitation value generated during the TC landfall processes.

In this study, we focus on the precipitation of the TC landfall process, namely the precipitation of 24 h before TC landfall and 24 h after TC landfall. For each TC event, we extract data on precipitation at 3 h intervals over a total duration these 48 h, thereby obtaining 17 sets of 3 h precipitation data records for each event. And we select the average precipitation values at − 3 h (3 h before TC landfall), 0 h (the moment of TC landfall), and 3 h (3 h after TC landfall) as representative of the peak precipitation when TC made landfall.

### Definition of variables

As mentioned earlier, the precipitation of TC increased before landfall and began to decrease after landfall. To quantify the precipitation characteristics of the TC processes, the precipitation changes are represented using the precipitation change rate before landfall (PrPCR) and the precipitation change rate after landfall (PoPCR). In this study, the PrPCR was defined as the slope of the linear fit to the average TCP from 24 h before landing (− 24 h) to the moment of landfall (0 h). The PoPCR was defined as the absolute value of the slope of the linear fit to the average TCP from the moment of landfall (0 h) to 24 h after landfall (24 h), PrPCR and PoPCR are calculated according to Eqs. (1) and (2), respectively.

1 $$ {\text{y}}_{1} = {\text{k}}_{1} {\text{x}}_{1} + {\text{b}}_{1} \left( { - 24 \le {\text{x}}_{1} \le 0} \right) $$

2 $$ {\text{y}}_{2} = {\text{k}}_{2} {\text{x}}_{2} + {\text{b}}_{2} \left( {0 \le {\text{x}}_{2} \le 24} \right) $$

in which *x*_1_ and *x*_2_ represent the time intervals before and after landfall, respectively, from the landing time of a TC. The time interval before landing is represented by a negative value, while, it is a positive value after landing; *y*_1_ and *y*_2_ are the average precipitation before and after landfall, respectively; k_1_ and k_2_ are the slopes of the linear fit, where k_1_ and ${ }\left| {{\text{k}}_{2} } \right|$ represents the PrPCR and PoPCR, respectively; and b_1_ and b_2_ are constants. We used the least squares method to fit the data and derive the values of parameters k_1_, k_2_, b_1_, and b_2_.

Besides, we assuming that precipitation changes are uniform, the durations of heavy precipitation after landfall and the total precipitation in 24 h can be calculated as follows:

3 $$ D = \frac{{R_{p} - r}}{{PCR_{a} }} $$

4 $$ R = \frac{{\left( {R_{24h} + R_{p} } \right)*24}}{2} $$

in which D and R represents the durations of heavy precipitation after landfall, namely the time of the precipitation decrease to a certain threshold *r*, and R represents the total precipitation in 24 h. In this study, the precipitation threshold *r* is set to 1 mm/h. $R_{p}$, $R_{24h}$ and $PCR_{a}$ represent the peak precipitation, the precipitation at 24 h, and PoPCR, respectively.

## Characteristics of TCP landings in China

### Changes in spatial pattern

**Figure 3:**
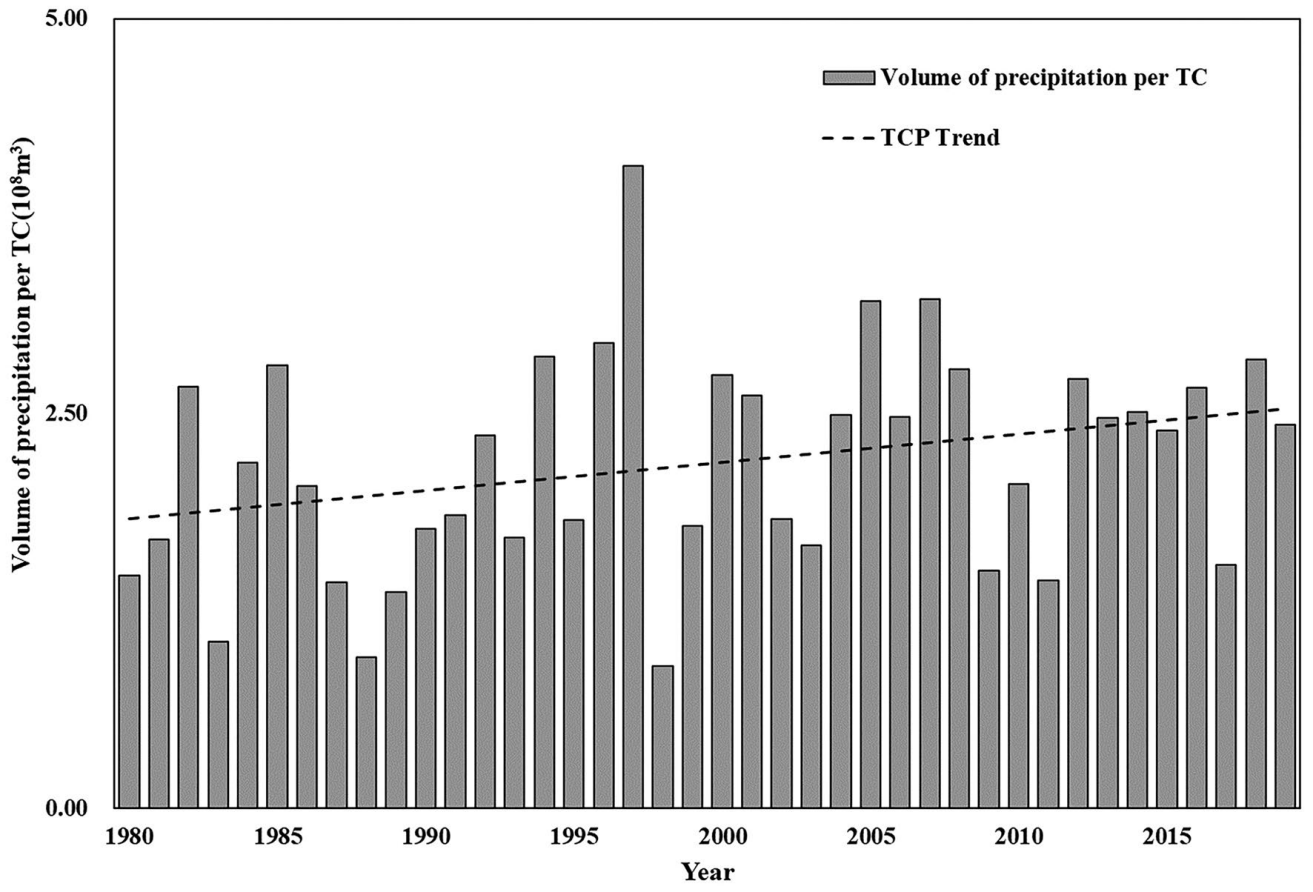
Total volume of precipitation per TC from 1980 to 2019.

The average volume of precipitation generated by TC every year from 1980 to 2019 in China was calculated according to the above method, and the results are shown in Fig. 3. The total volume of precipitation per TC showed an overall increasing trend from 1980 to 2019, and highest in 1997.

**Figure 4:**
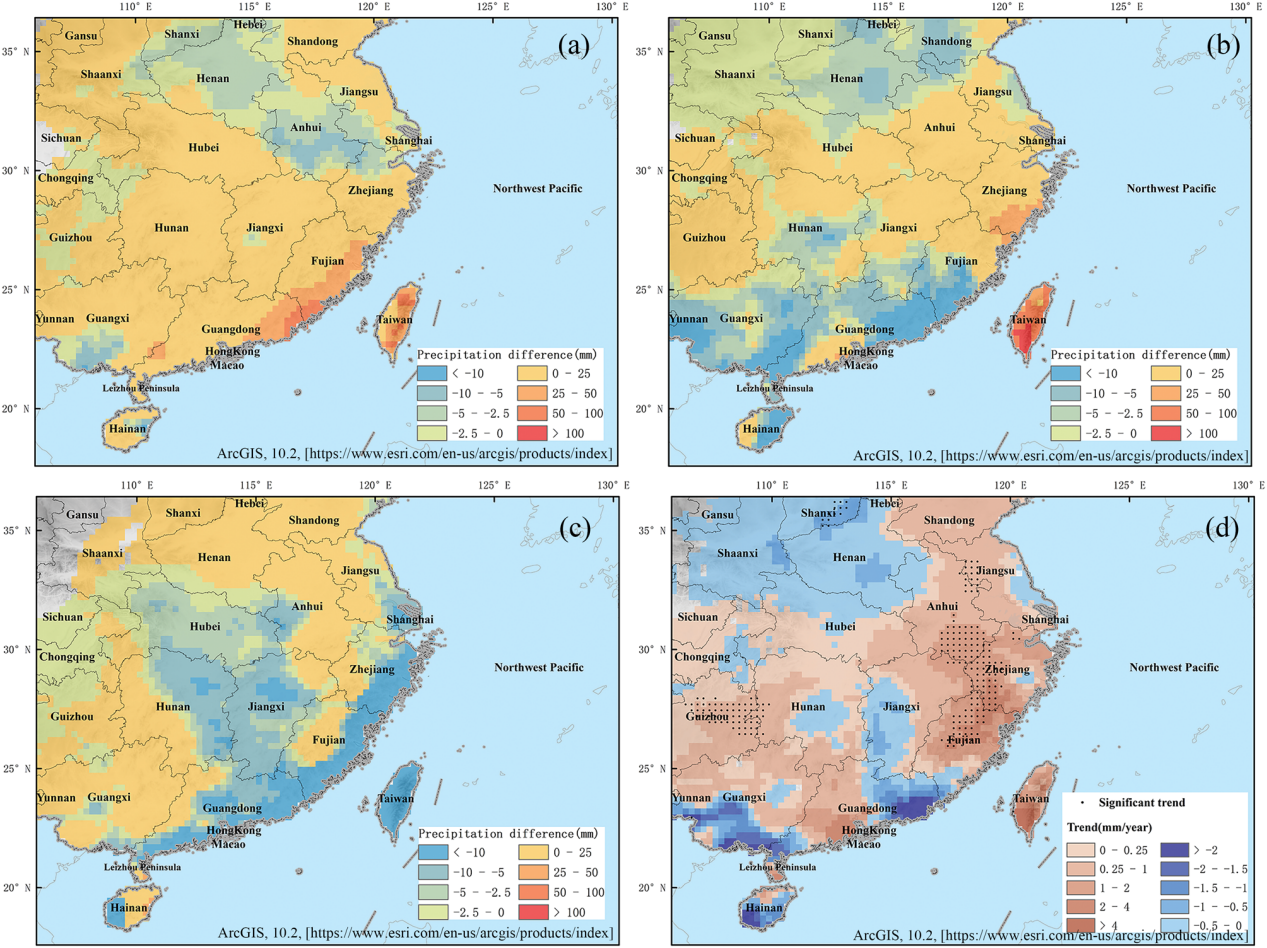
Spatial characteristics of TCP along the southeast coast of China in different periods from 1980 to 2019. (**a**) The difference in annual average precipitation between the 1980s and1990s. (**b**) The difference in annual average precipitation between the 1990s and 2000s. (**c**) The difference in annual average precipitation between the 2000s and 2010s periods. (**d**) Trends in TCP changes from 1980 to 2019. 1980s:1980–1989; 1990s:1990–1999; 2000s:2000–2009; 2010s:2010–2019.

In China, most TCs make landfall in the southeastern coastal areas, including Hainan, Guangdong, Fujian, Zhejiang, Jiangsu, and Shanghai. The southeastern region of China is one of the areas severely affected by TCR along the northwest Pacific coast. To analyze the changes in precipitation associated with TCs in the southeastern coast of China, the differences in precipitation for adjacent 10-year periods from 1980 to 2019 and TCP change trend were calculated (Fig. 4). 1980s, 1990s, 2000s and 2010s represent the four periods: 1980–1989, 1990–1999, 2000–2009, and 2010–2019, respectively. Compared to that in the 1980s, the TCP in most areas along the southeastern coast of China significantly increased during the 1990s (Fig. 4a). Compared to the 1990s, Taiwan, part of Guangdong and areas from 25° N to 32° N except southern part of Hunan experienced a significant increase in TCP during 2000s (Fig. 4b). However, there was a relatively larger proportion of areas in the southern region with a decreasing TCP (Fig. 4b). Compared to that in the 2000s, the TCP in China in the 2010s decreased in most coastal areas and increased in most inland areas; this may imply that during the 2010s, the impact of precipitation after TC landfall increased (Fig. 4c). From 1980 to 2019, except for the northeastern part of Guangdong, the southern part of Guangxi, and Hainan, the TCP along the coast of China showed an increasing trend (Fig. 4d). Among them, Fujian and Zhejiang showed the most significant increase in TCP, passing the significant test at significance level α = 0.1(Fig. 4d).

### TC process precipitation

**Figure 5:**
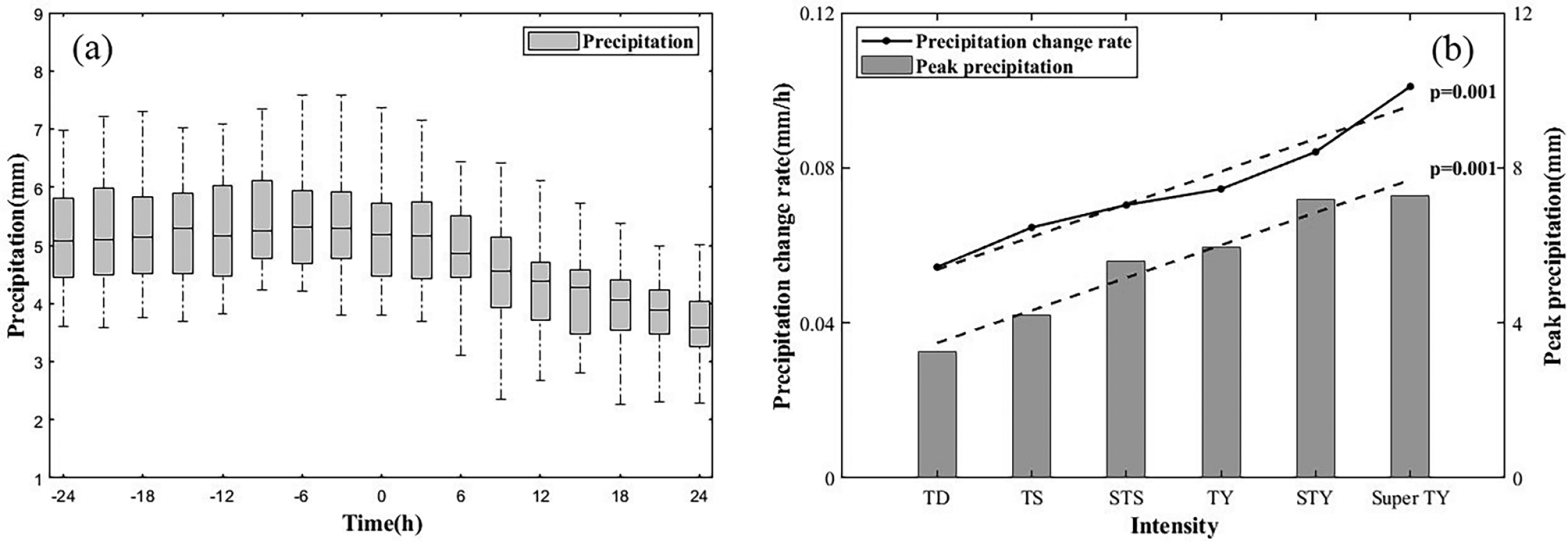
(**a**) TCP box plot of 327 TCs from 1980 to 2019 during landfall (the x-axis represents the time difference from the landing moment (0 h), and the y-axis represents the TCP). (**b**) Peak precipitation and PoPCR of TCs under different landfall intensities.

According to the above method, the precipitation of each TC that made landfall in China from 1980 to 2019 was extracted. A total of 327 TCs made landfall in China from 1980 to 2019, including 67 TCs that passed through Taiwan and then landed on the mainland of China again. Figure 5a shows the distribution of TCP quantity at each time points during the landfall process over the span of 40 years. In Fig. 5a, it can be found that there was a turning point in the change in precipitation around the moment of TC landfall. The TCP of landed TC slightly increased within 24 h before landfall, followed by a significant decrease within 24 h after landfall. The PrPCR is concentrated at approximately ± 0.04 mm/h, and the PoPCR is within the range of 0–0.15 mm/h.

Figure 5b shows the average PoPCR and peak precipitation were calculated for different grades of TCs. It can be seen that the peak precipitation when TC landfall and PoPCR are strongly correlated with its intensity (*p* < 0.05) at the moment of landfall. Higher intensity of a TC is when it makes landfall predicts higher peak precipitation levels subsequently, and a more rapid decline in precipitation intensity after landfall.

**Figure 6:**
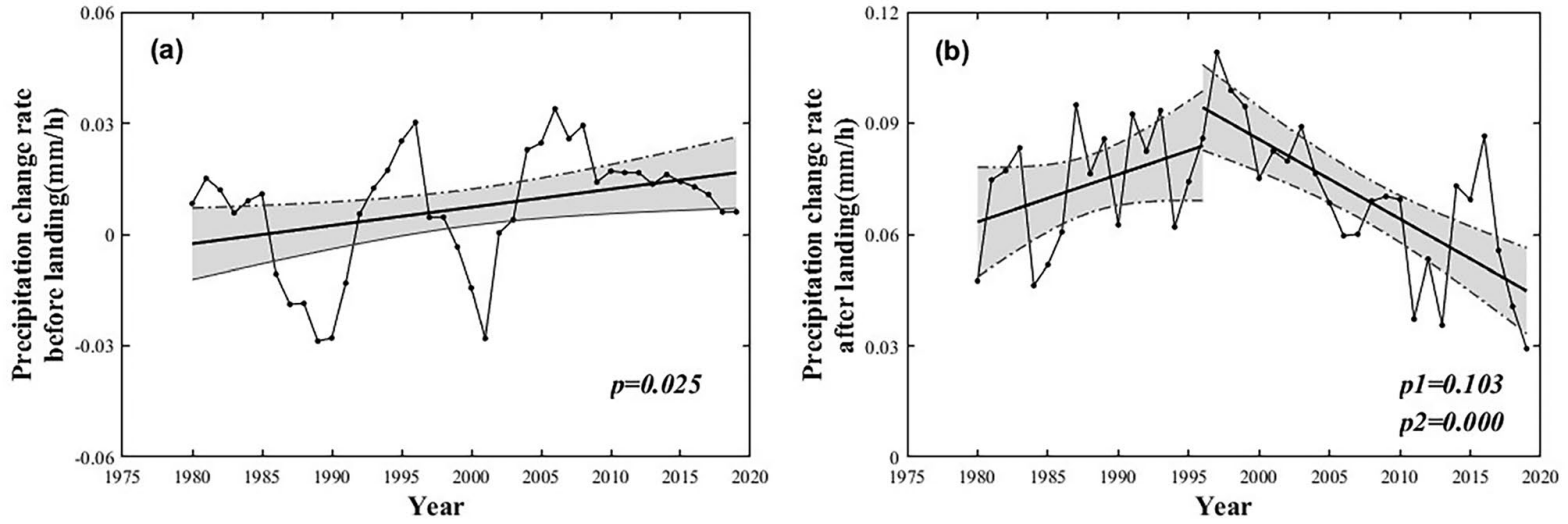
Trend analysis of the (**a**) PrPCR and (**b**) PoPCR. The precipitation change rate before landfall significantly increased from 1980 to 2019. The *p* value is 0.025. The precipitation change rate after landfall has significantly decreased since 2000. The *p* value is 0.000.

The interannual variations in PCR during the TC process were analyzed. From 1980 to 2019, an average of approximately 8 TCs made landfall in China every year. In this study, the Mann‒Kendall (M–K) trend test method with a 95% confidence interval was used to test whether there were temporal trends in three variables: the PrPCR, the peak precipitation, and the PoPCR. The results (Fig. 6) indicated that the PrPCR increased significantly from 1980 to 2019 (Fig. 6a). PoPCR exhibited two different change periods, with a slight increase but no significant change from 1980 to 1999 and a significant decrease from 2000 to 2019 (Fig. 6b). Over the past 40 years, there has been no significant trend for peak precipitation, which fluctuates approximately 5 mm.

The PrPCR of TCs increased significantly from 1980 to 2019, with a decreasing proportion of negative values. This change may be related to the significant increase in global sea surface temperatures and TC intensity over the past few decades^13–16^. The increase in PrPCR implies that the rainfall triggered by TCs intensifies within 24 h before landfall. The rate at which precipitation decreases after landfall is closely related to the impact of TCP on land. Under the same peak precipitation conditions, the more slowly precipitation decreases after landfall, the greater flood or landslide caused by TCP. As shown in Fig. 6b, the PoPCR has significantly decreased since 2000, which indicates that TCP will take longer time to decrease to a low level. Hence, attention must be devoted to TCP.

In summary, since 1980, the PrPCR has increased significantly, while the peak precipitation caused by landed TCs in China has remained relatively stable. Since 2000, the rate of decrease in precipitation intensity after landfall has slowed significantly, and there has been an overall increasing trend in total precipitation within 24 h before and 24 h after the landfall of TCs, which is consistent with the conclusions of other studies^16,19,20,24^.

### Regional characteristics

**Figure 7:**
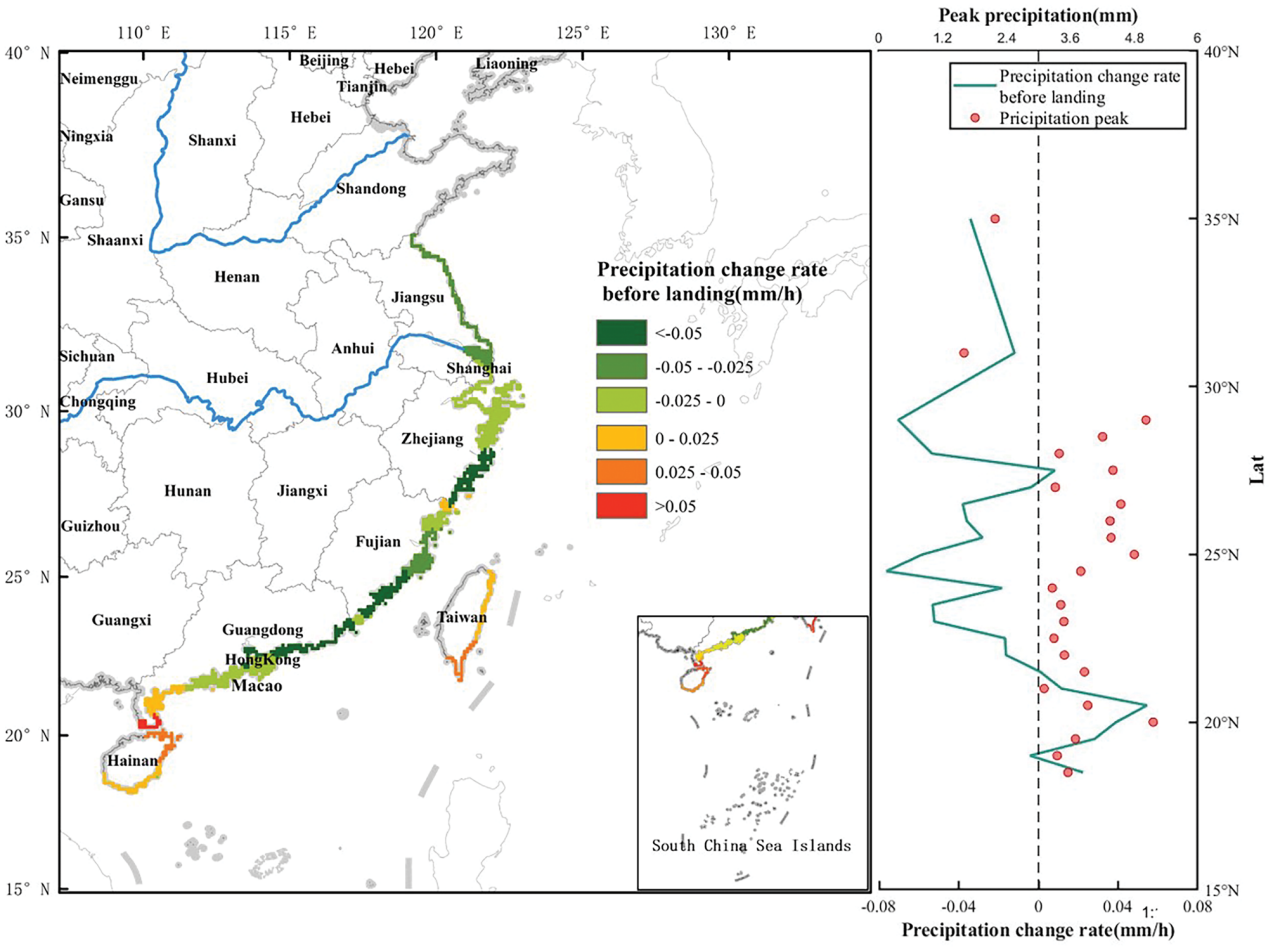
PrPCR distribution of TCs at different latitudes. The left coastline color band (negative values in green and positive values in red) represents the data corresponding to the PrPCR, as depicted in the right line graph. The red points indicate the peak precipitation.

**Figure 8:**
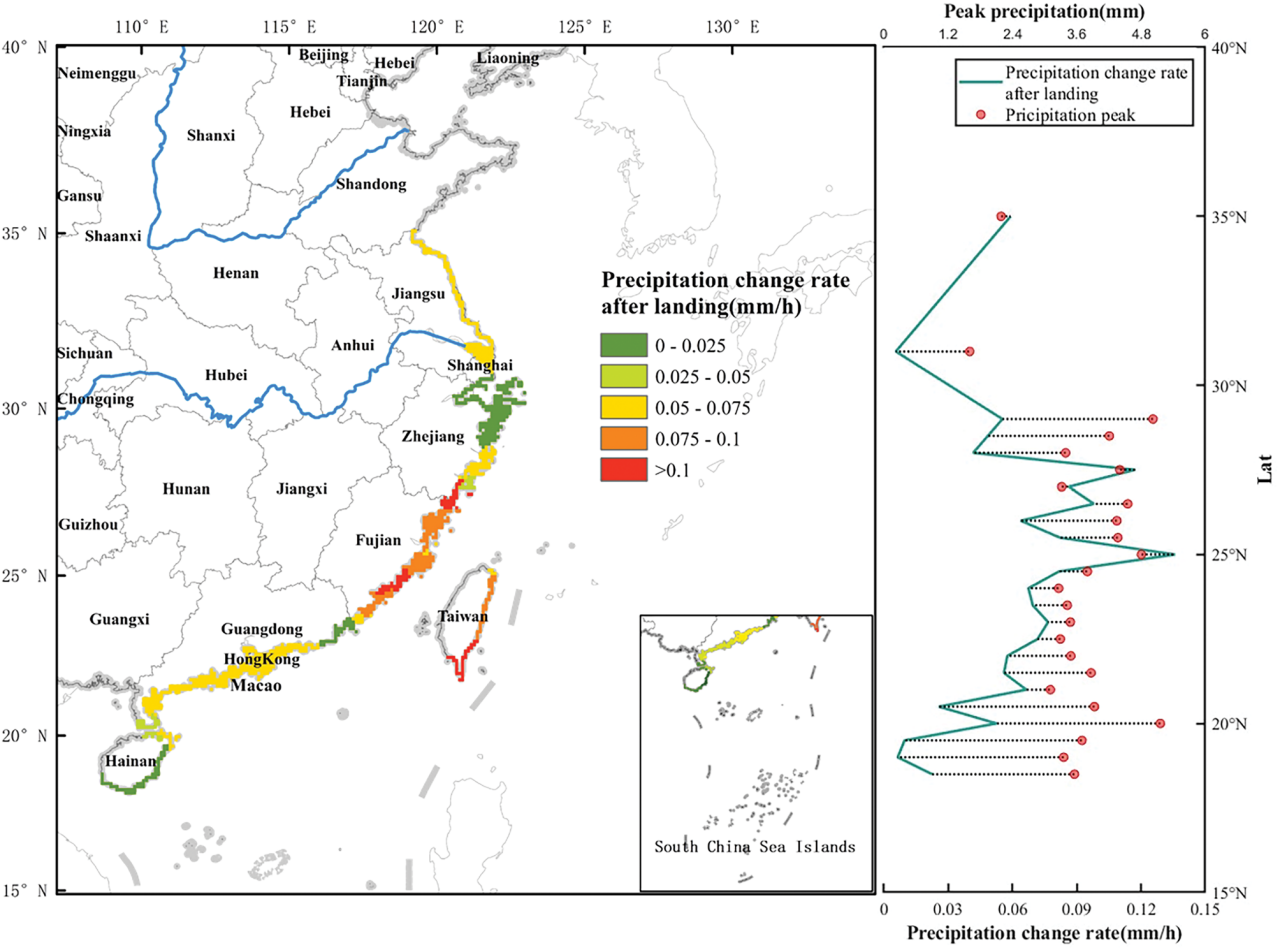
PoPCR distribution of TCs at different latitudes. The left coastline color band represents the data corresponding to the PoPCR, as depicted in the right line graph. The red points indicate the peak precipitation.

To investigate the variability characteristics of TCP in different regions, the PoPCR and peak precipitation at various latitudes were calculated. From 1980 to 2019, the TCs that made landfall in China were mainly concentrated between 18° N and 35° N. Among them, more than 50% made landfall at the south of 23° N. The higher the latitude is, the fewer landfalls there are. Figures 7 and 8 show the PrPCR and PoPCR distributions, respectively, with a latitudinal interval of 0.5°.

Figure 7 shows that the average precipitation in most areas within 24 h before the landfall of a TC began to decrease (with a negative PrPCR), but this change was not obvious. The PrPCR was concentrated at approximately ± 0.06 mm/h. 22° N is the turning point from positive to negative PrPCR. Regions such as Hainan and the Leizhou Peninsula (south of Guangdong) showed an increasing trend in precipitation before TC landfall. In addition, there are also a few areas in the northern part of Fujian with positive PrPCR results before landfall. After a TC makes landfall, the decreasing rate of precipitation after landfall shows a general trend of first increasing and then decreasing from south to north along the southern coast of China. The maximum value was reached near the coast of Fujian. The peak precipitation is concentrated between 25° N and 29° N.

**Figure 9:**
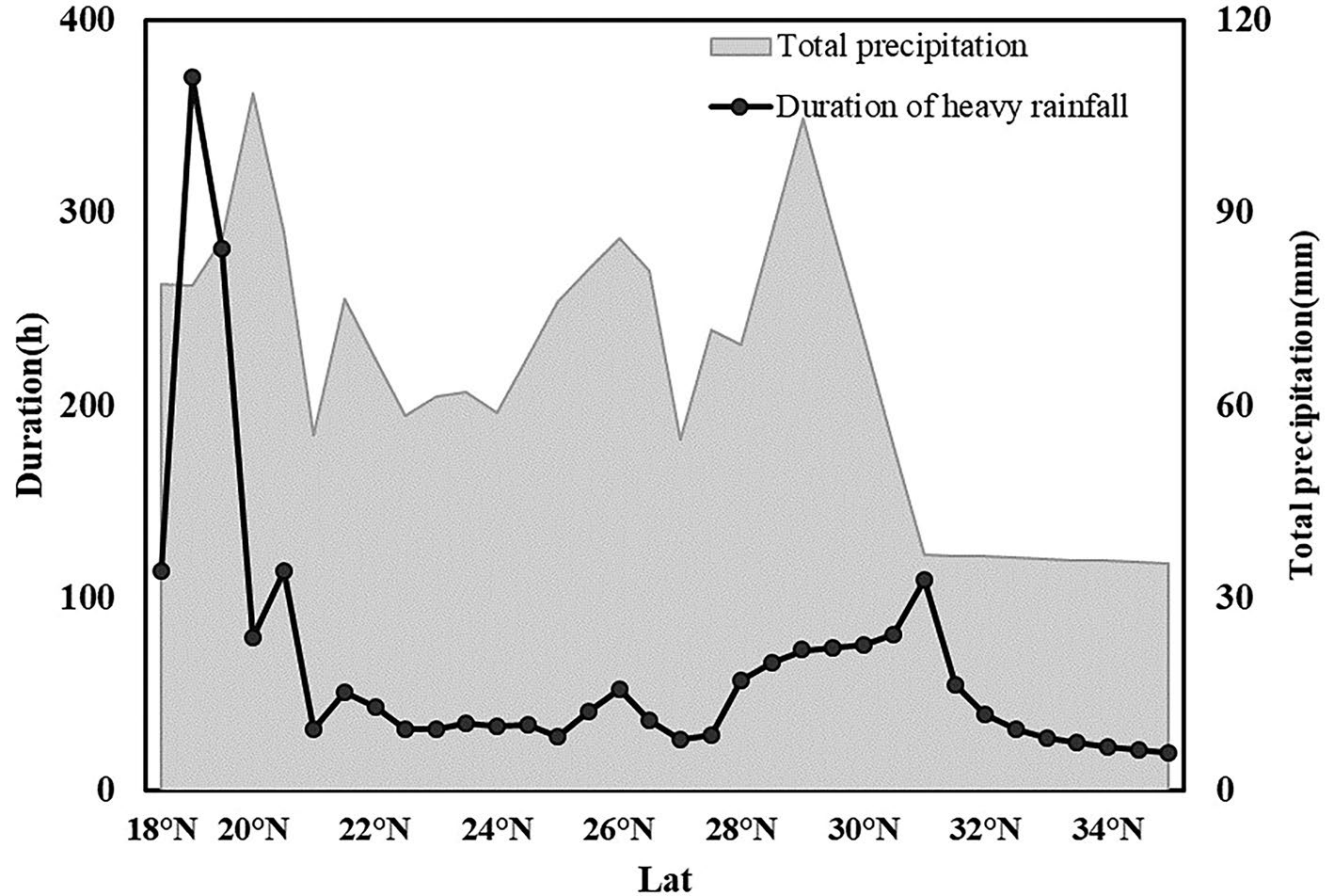
Duration of heavy rainfall (filled graph) and total precipitation (black line) with latitude within 24 h after TC landfall. Assuming that the precipitation change after TC landfall is uniform, this data is calculated using the peak precipitation and the PoPCR.

When the peak precipitation of a TC is high and the rate of decrease in precipitation after landfall is relatively slow, the total precipitation caused by the TC is greater, and the precipitation lasts longer. Therefore, it is necessary to pay special attention to areas with large peak precipitation and slow PoPCRs. In this work we measured the severity of the impact of TCP on a region from two aspects: total precipitation and duration of precipitation. The durations of heavy precipitation after landfall and the total precipitation in 24 h were calculated according to the peak precipitation and the PoPCR (Fig. 9). Figure 8 shows that the precipitation during the landfall of the TC in Hainan was characterized by high peak precipitation and low PoPCR. Figure 9 also indicates that the heavy precipitation and total precipitation in Hainan are greater than those in other areas. Furthermore, considering the increasing trend in precipitation during the 24 h before landfall of a TC (Fig. 7), Hainan is at high risk of being affected by heavy precipitation during the 24 h before and after landfall. In addition to Hainan, which has a range of [28° N, 30° N] and includes southern Zhejiang and parts of Shanghai, the ideal total precipitation is also very high within 24 h after landfall, and the ideal duration of heavy rainfall is also very long. The impact of the TCP is also severe in this area. The northern coasts of Fujian and Guangdong have experienced large and concentrated landed TC in the past 40 years, but the impact of TCP has been less severe than that in these two regions. This may be because most TCs make a secondary landfall in this region, and they are initially weakened by the blocking effect of Taiwan. Precipitation has already declined faster before landfall, and it has declined faster after landfall. As a result, the duration of heavy precipitation associated with the TC was relatively short, but the peak precipitation amount remained high. The occurrence of short-term intense precipitation should not be ignored.

**Figure 10:**
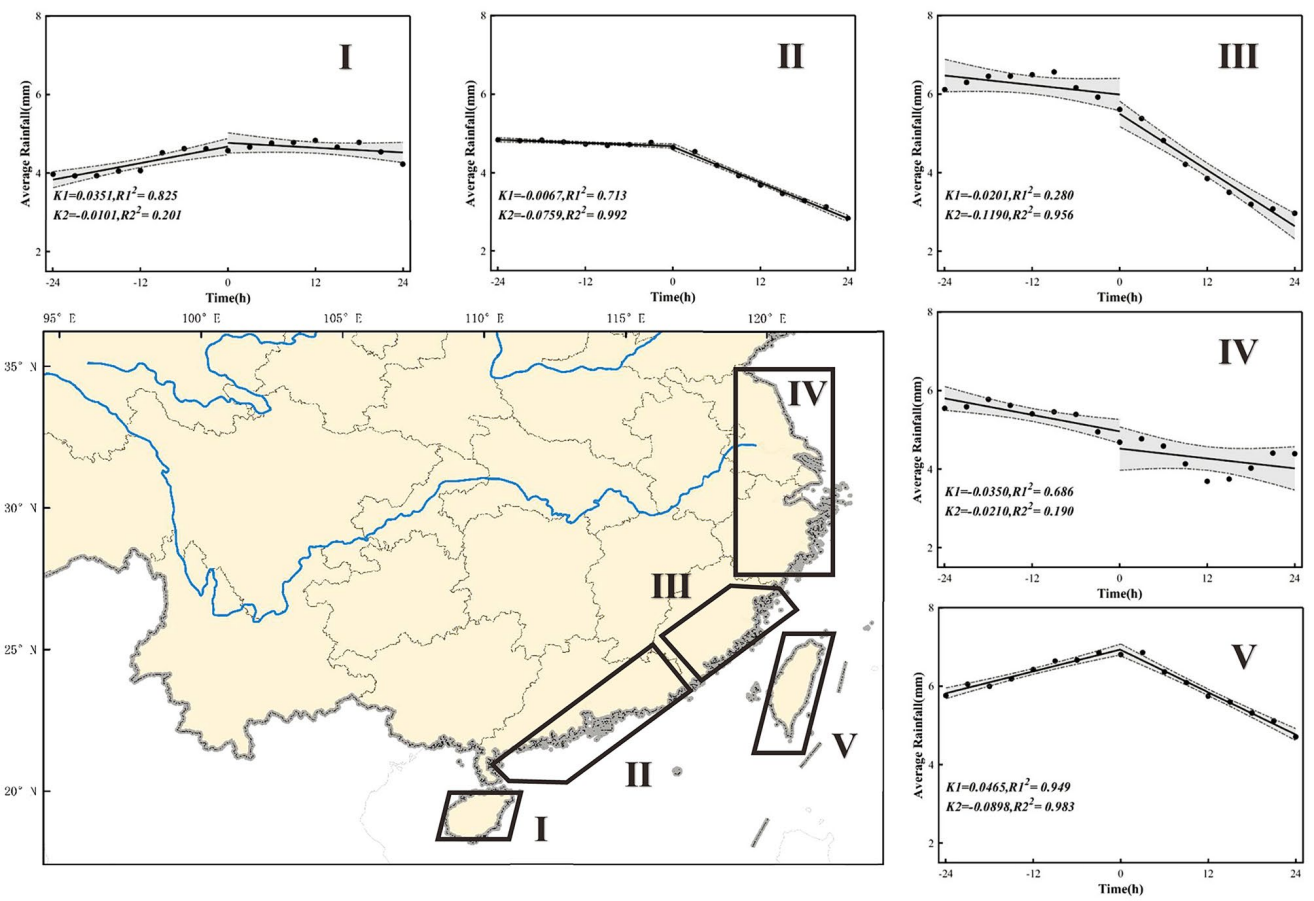
Segmented fitting graph of precipitation changes within 24 h before and after the landfall of TCs in various regions. . I, II, III, IV, V respectively represent the coastal areas of Hainan, Guangdong, Fujian, Zhejiang Jiangsu and Shanghai, and Taiwan. The segmented linear fitting results are listed in Table 1.

According to the above precipitation characteristics of TCs landing at different latitudes, combined with administrative divisions and sample sizes, the study area is divided into five regions, as shown in Fig. 10. Region I includes the coast of Hainan, Region II includes the coast of Guangdong, Region III includes the coast of Fujian, Region IV includes the coasts of Zhejiang, Jiangsu, and Shanghai, and Region V covers Taiwan. Table 1 lists the three variables, PrPCR, peak precipitation, and PoPCR, in the five regions from 1980 to 2019.

**Table 1:** Statistical analysis of precipitation in different regions 24 h before and after the landfall of a TC (“*” indicates passing the significance test with a significance level of 0.05).

Region	PoPCR (R^2^, *p* value)	Peak precipitation (mm)	PoPCR (R^2^, *p* value)
I	0.0351 (0.825, 0.000)	4.619	− 0.0101(0.201, 0.227)
II	− 0.0067 (0.713, 0.004)	4.655	− 0.0759 (0.992, 0.000)
III	− 0.0201 (0.280, 0.143)	5.639	− 0.1190 (0.956, 0.000)
IV	− 0.0350 (0.686, 0.006)	4.803	− 0.0210 (0.190, 0.240)
V	0.0465 (0.949, 0.000)	6.840	− 0.0898 (0.983, 0.000)
All regions	0.0065 (0.355, 0.090)	5.233	− 0.0692 (0.984, 0.000)

The TCs that made landfall in Region I and Region V had similar precipitation change patterns. The precipitation during the 24 h before landfall showed an upward trend and reached its peak around the time when the TC made landfall. After landfall, the amount of precipitation starts to decrease. However, the rate of precipitation decreases in region I is slower than that in region V. This might be because the TCs in region I have more westward tracks and do not go deep inland after landfall. As a result, the TCs in region I maintain a relatively high precipitation intensity within 24 h before and after landfall. Compared to those in regions I and V, the precipitation intensities of TCs in regions II, III, and IV exhibited a downward trend earlier. However, in region III, the precipitation intensity rate decreased faster after landfall. This may be because TCs in region III were strongly blocked by Taiwan during the early stage of landfall. The precipitation in Region I lasts relatively long, so attention should be given to preventing precipitation after the landfall of TCs. From the perspective of peak precipitation, TCs in region V have the highest peak precipitation. Although precipitation decreases rapidly after landfall, the intensity of precipitation is still very high within 24 h after landfall, and the impact of precipitation cannot be ignored. TCs in region III also have relatively large peak precipitation.

## Conclusion and discussion

In this study, we utilized IBTrACS TC track data and MSWEP precipitation data to extract time series of regional average precipitation every 3 h within 24 h before and after the landfall of TCs in China from 1980 to 2019. The PrPCR, peak precipitation, and PoPCR were calculated for the TCs to quantify the TCP. The interannual and regional characteristics of precipitation changes during the landfall of TCs in China were analyzed. The obtained parameters represent the variation in precipitation during the TC process. The results show the following.

The average precipitation during the landfall of TCs in China generally has the characteristics of being relatively stable before landing and declining after landing. The maximum precipitation often occurs around the moment of landfall during its landfall process, and it rapidly decreases after landfall. The precipitation during the process slowly increased before landfall and rapidly decreased after landfall. Variables such as PoPCR are closely related to the intensity of landed TC.

From a temporal perspective, since 1980, the peak precipitation of TCs that made landfall in China has been relatively stable, and the PrPCR has increased significantly before landfall. Since 2000, the PoPCR has decreased significantly, which implies that the total precipitation generated within 24 h after the landfall of a TC shows an overall increasing trend. Consequently, the negative impacts resulting from TCP will be further enhanced in the future.

In terms of spatial distribution, the precipitation of TCs that land in different regions shows different characteristics. Regions such as Hainan, southern Zhejiang, and some areas in Shanghai have high peak precipitation and a slow decrease after landfall. The duration and total precipitation values are greater than those in other regions. Therefore, it is necessary to focus on preventing disasters caused by TCP in these areas.

The above analysis from different perspectives reveals the characteristics of precipitation changes during the process of TC landfall. The landed TC shows an increase in the duration and intensity of precipitation. In the future, it is necessary to focus on both short-term heavy rainfall and long-term sustained precipitation when preventing TCs.

This study provides a reference for selecting parameters related to the variations in precipitation modeling during the TC process. Regions can take appropriate preventive measures based on the characteristics of precipitation changes to reduce the losses caused by TCP. This study also has certain limitations. Although the extraction results of TCP were compared and analyzed with different radius thresholds and minimum precipitation intensity thresholds, due to the strong uncertainty of TCP, the precipitation extraction process may neglect peripheral rainfall or include precipitation from other weather systems. However, the precipitation extraction method still needs improvement. Analyzing the changes in precipitation during TC processes using regional average rainfall can effectively express the changing trends of precipitation, but the extremes of TCP will also be ignored. In future research, variables such as maximum rainfall can be added for comparative analysis. In future studies, other TC paths and precipitation data can be obtained in addition to the IBTrACS and MSWEP data for comprehensive analysis to reduce the uncertainty of the research results. In addition, the application of parameters related to the variations in precipitation during the TC process in TCP simulations must be explored.

## Data Availability

Best track data come from the International Best Track Archive for Climate Stewardship (IBTrACS): https://www.ncei.noaa.gov/data/international-best-track-archive-for-climate-stewardship-ibtracs/v04r00/access/csv/. Precipitation data come from the Multi-Source Weighted-Ensemble Precipitation (MSWEP): https://www.gloh2o.org/mswep/.
